# He Votes or She Votes? Female and Male Discursive Strategies in Twitter Political Hashtags

**DOI:** 10.1371/journal.pone.0087041

**Published:** 2014-01-29

**Authors:** Evandro Cunha, Gabriel Magno, Marcos André Gonçalves, César Cambraia, Virgilio Almeida

**Affiliations:** 1 Computer Science Department, Federal University of Minas Gerais, Belo Horizonte, Minas Gerais, Brazil; 2 College of Letters, Federal University of Minas Gerais, Belo Horizonte, Minas Gerais, Brazil; University of Warwick, United Kingdom

## Abstract

In this paper, we conduct a study about differences between female and male discursive strategies when posting in the microblogging service Twitter, with a particular focus on the hashtag designation process during political debate. The fact that men and women use language in distinct ways, reverberating practices linked to their expected roles in the social groups, is a linguistic phenomenon known to happen in several cultures and that can now be studied on the Web and on online social networks in a large scale enabled by computing power. Here, for instance, after analyzing tweets with political content posted during Brazilian presidential campaign,we found out that male Twitter users, when expressing their attitude toward a given candidate, are more prone to use imperative verbal forms in hashtags, while female users tend to employ declarative forms. This difference can be interpreted as a sign of distinct approaches in relation to other network members: for example, if political hashtags are seen as strategies of persuasion in Twitter, imperative tags could be understood as more overt ways of persuading and declarative tags as more indirect ones. Our findings help to understand human gendered behavior in social networks and contribute to research on the new fields of computer-enabled Internet linguistics and social computing, besides being useful for several computational tasks such as developing tag recommendation systems based on users' collective preferences and tailoring targeted advertising strategies, among others.

## Introduction

Language, as a major cultural trait shared by societies, is known to be an important element which reflects values and roles played by individuals in the communities that they belong to. Social factors of all sorts, such as gender, age, level of education, socioeconomic class and many others influence the way it is used, and the study of these factors is a relevant task in sociolinguistics, a field of linguistics focused on the relations between language and society [1]–[3].

One of these relevant social factors is undoubtedly the gender of the speakers: it is known that men and women express themselves differently, reflecting the behavior patterns associated with their roles in the social groups. Many studies have already correlated gender to linguistic variation and significant differences between lexicon, pronunciation, morphology, syntax, speech organization and language interaction of female and male speakers have been found in the last 50 years [1]–[3]. Naturally, differences in the linguistic behavior between genders may vary from society to society, since the roles played by individuals of each gender across distinct communities are also different.

One of the first studies that correlated gender to linguistic variation examined the pronunciation of the final *-ing* in the Boston area [4]. It was found a significant difference between the pronunciation of female and male speakers, which was confirmed by studies of the same linguistic variable in British and Australian communities, with similar results [5], [6]. Many other studies also showed several contrasts between the way men and women use language, including in Brazilian Portuguese [7], the language in which we focus in this paper. Most of them point out that female speakers are more likely to use prestige variants than male speakers. This characteristic was found not only in English, but also in other Western modern languages. Other studies, however, indicated that this pattern is different, for instance, in some Indian and Islamic communities, where prestige variants are usually predominant not among women, but among men [1]. These results support that the correlation between gender and linguistic variation must be associated with the social organization of the studied communities - since, as formulated by Simone de Beauvoir, ''one is not born, but rather becomes, a woman'' [8].

It is possible to list a number of other gender-related linguistic and discursive patterns, keeping in mind that these patterns are just reflections of socio-cultural situations, not biologically determined. Past studies that examined characteristics of female and male linguistic behaviors found contrasts in relation to questions and responses, turn-taking, topic change, self-disclosure and many others. It has been argued that Western women's communication patterns are distinct from those of men not only in form, but also in content, and that female speech often reflects the socialization of women into subordinate roles in patriarchal societies [9]. Some interesting results, related to a vision of societies being polarized by two forces - power and solidarity -, were found with respect to the use of pronouns in many modern languages [10]. According to this perspective, in most communities - including in Latin America [11] - there is an asymmetrical power relationship between men and women that determines which pronouns should be used in each communicative situation so that traditional hierarchical differences will be maintained; on the other hand, speakers of the same social rank are considered to occupy similar positions, and therefore they use pronouns that express a relation of mutual identification and solidarity.

The development of online social networks and, consequently, the increase of user-generated content on the Web raised the capacity of conducting a number of studies in the fields of humanities, leading to a better comprehension of various social phenomena - including political elections [12]–[14] - and pose a very interesting question: do language differences between men and women manifest themselves also in online interaction? If so, to what extent and in which situations does this phenomenon occur? In this article, our aim is to verify the existence of differences between female and male discursive strategies when performing a specific and increasingly common task on the Web: participating in politics by designating hashtags to tweets carrying a political message.

## Materials and Methods

### Data

With the aim of performing the experiments proposed here, we used a dataset collected by the Brazilian National Institute of Science and Technology for the Web, that runs a Twitter API to obtain data about specific topics with the purpose of presenting what kind of content and information is circulating on the Web, in a project called The Web Observatory (see http://www.observatorio.inweb.org.br/english.html for additional details about the project and the data collection).

The complete dataset contains all 9,789,596 public tweets - including hashtags, when existing - regarding the 2010 Brazilian elections from March 2 to December 17, in addition to public personal information from all users that posted these tweets, including their given names. A comprehensive description of Twitter and hashtags, which are central elements in this study, can be found in https://support.twitter.com/groups/50-welcome-to-twitter. For ethical reasons, no attempts were made to obtain access to information set as private. This dataset is publicly available and may be obtained from the corresponding author upon request.

**Figure 1:**
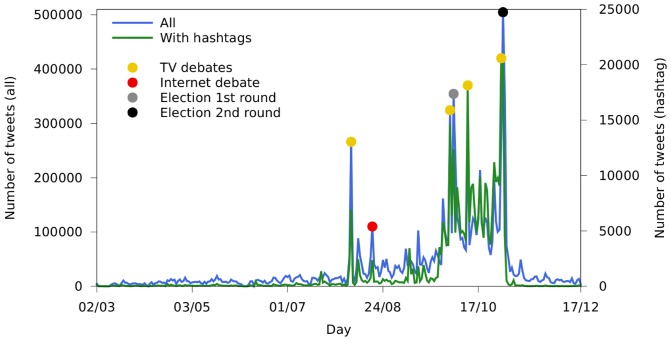
Evolution of the activity related to the 2010 Brazilian elections on Twitter.

Figure 1 exhibits the evolution of Twitter activities related to the 2010 Brazilian presidential elections. It shows, on a daily basis, the total number of tweets and the number of tweets which include hashtags: as expected, the activity largely increases in key moments. It is worth noting the small peak on the day of the first Internet-held presidential debate in Brazilian history.

### Methods

#### Gender Inference

In order to implement the analyses, it was initially necessary to infer the gender of the users, since this information is not present in Twitter profiles - because there is no field ''gender'' to be filled in by the network members. Users are only required to fill in a field ''''full name''.

**Figure 2:**
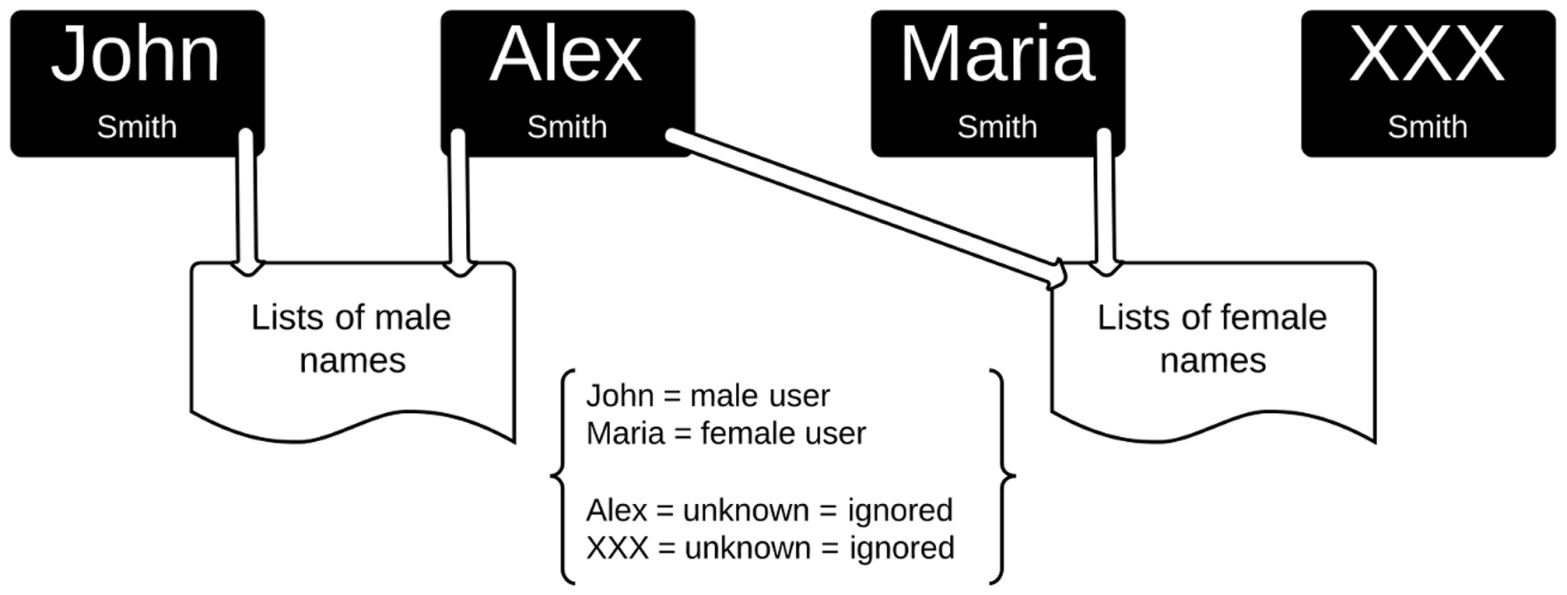
Process of gender inference adopted in this study. According to Burger et al. [15], the method of comparing given names on profiles to lists of predefined gendered names has an accuracy of 89.1% for discriminating gender on Twitter.

The task of gender inference was performed by the simple comparison of the first names provided in the field ''full name'', and thus available in the users' pages, with lists of female and male first names in Portuguese, extensively found in Internet, such as in http://www.listadenomes.com.br/. According to Burger et al. [15], this method provides an accuracy of 89.1% in the specific case of Twitter. Names considered unisex or gender-neutral were ignored. We also ignored users whose first names were missing from the lists adopted. In summary, we were able to retrieve the gender of 459,231 users, authors of 3,395,332 tweets (34.7% of the complete dataset): 243,220 are men, 216,011 are women. Figure 2 depicts the process of gender inference adopted in this study.

#### Collections of Hashtags

**Figure 3:**
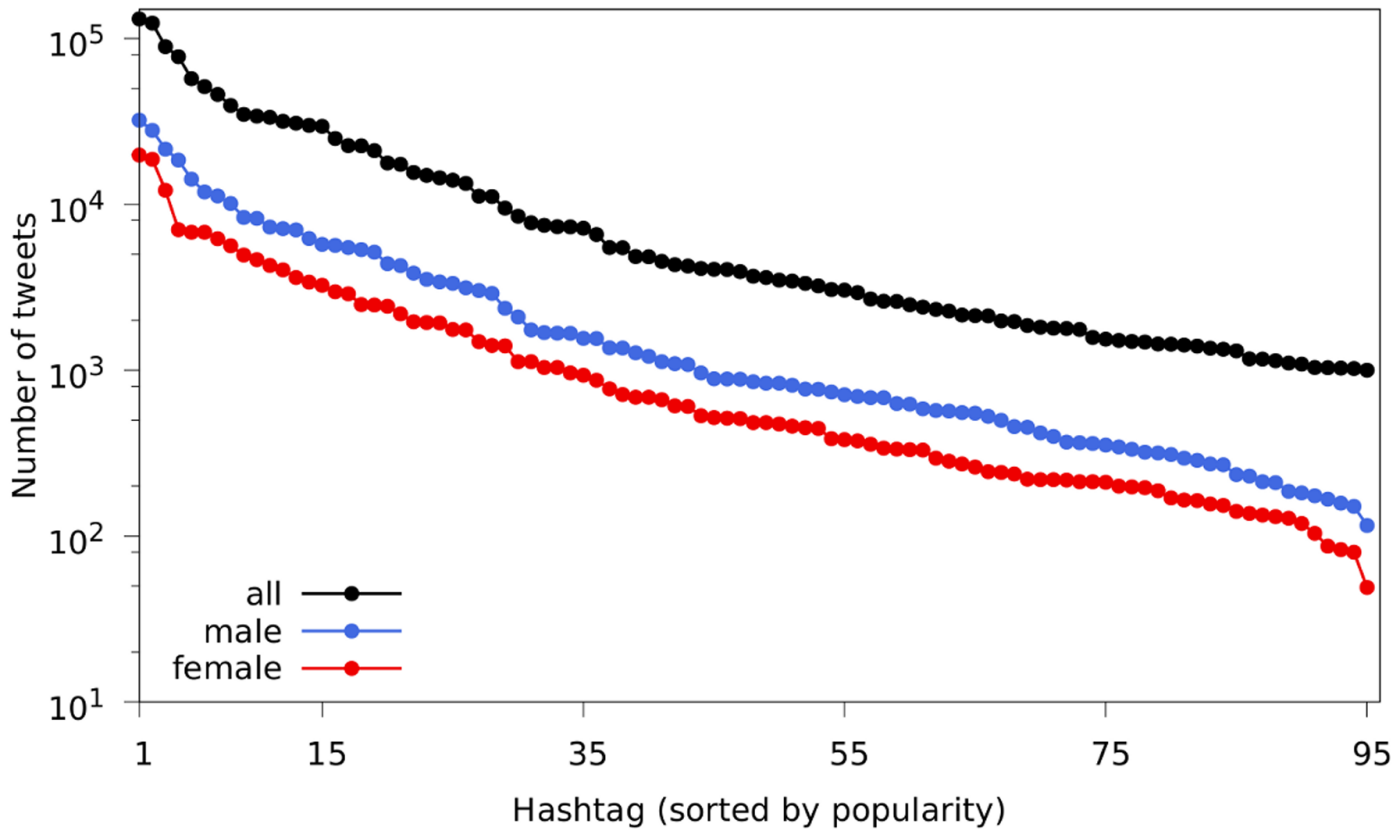
Frequency of usage of the hashtags vs. their positions in a popularity ranking. As shown in previous investigations [16], a few hashtags are very popular, while most of them are not widely employed by network members.

In the following step, only tweets containing hashtags were selected, totaling 355,171 messages (10.5% of the dataset after the gender inference), and the 95 different tags that appeared in at least 1,000 of these messages were collected. Figure 3 shows the frequency of usage of these hashtags and supports previous research indicating that few tags are used in most of the tweets, while the majority of them appear in only a few posts [16].

These 95 hashtags were further divided into four subdatasets built manually and according to the personal analysis of the authors ourselves, who examined all the tags and categorized them in one of the subdatasets. The goal of this division is that we aim to investigate the users' choices of tags expressing particular points of view related to the main subject of the messages. Therefore, we created subdatasets formed by hashtags associated to the following subtopics: support to Dilma Rousseff (29.5% of tags); support to José Serra (10.5%); opposition to Dilma Rousseff (11.6%); and opposition to José Serra (14.7%). Dilma Rousseff - woman - and José Serra - man - were the main candidates in the 2010 Brazilian presidential elections. It is important to clear out that not necessarily a hashtag opposing a candidate will be supporting the other one: for instance, #dilmanunca (''Dilma never'') is opposing Dilma, but not clearly and directly supporting Serra. Hashtags considered neutral, neither supporting nor opposing any candidate, such as #eleicoes (''elections'') and #votabrasil (''vote Brazil''), or supporting or opposing minor candidates, as in #votemarina (''vote for Marina Silva''), were ignored in this phase. They represent 33.7% of the totality of hashtags considered. Table 1 shows some examples of hashtags which are part of the subdatasets considered in this paper.

**Table 1:** Examples of hashtags which form the subdatasets built.

Support to Dilma Rousseff (total = 28 tags)	Support to José Serra (total = 10 tags)
#dilma13 (''Dilma 13 [Dilma's number]'')	#serra45 (''Serra 45 [Serra's number]'')
#votodilma (''I vote for Dilma'')	#votoserra (''I vote for Serra'')
#dilmapresidenta (''Dilma for president'')	#br45il (''Br45il'')
#soudilma (''I am Dilma'')	#45confirma (''confirm 45'')

Opposition to Dilma Rousseff (total = 11 tags)	Opposition to José Serra (total = 14 tags)
#forapt (''PT [Dilma's party] out'')	#forapsdb (''PSDB [Serra's party] out'')
#dilmamente (''Dilma lies'')	#serramilcaras (''Serra one thousand faces'')
#dilmanao (''no Dilma'')	#serranao (''no Serra'')
#dilmafujona (''Dilma fugitive'')	#serracaluniador (''Serra slanderer'')

Although our datasets were manually built in this particular study, automatic methods based on supervised machine learning techniques [17] could be used to classify tweets with hashtags, not only regarding whether they were related to political discussions, but also to infer the ''polarity'' of these tweets, with reasonable accuracy [18]. However, as a first exploratory study, we preferred to rely on the manual assessment to guarantee high precision and coverage, leaving the use of such automated tools for next studies. Moreover, having manually labeled all these tags, our collection now becomes an important asset to evaluate the effectiveness of these automatic tools in future investigations.

In the whole dataset, 55.9% of the users posted exactly one type of hashtag, even if more than once; 21.2% of them posted exactly two types of hashtags; 8.8% posted three types; and 14.1% of the users in the dataset posted four or more different types. These numbers are comparable across the different subdatasets: respectively, 74.0% and 74.7% of the supporters of Dilma Rousseff and José Serra used less than 10% of the hashtags belonging to the correspondent subdataset; 87.8% and 91.5% of them used less than 20%; and 93.4% and 95.8% used less than 30%. It shows that, in general, users tend to employ a limited number of hashtags in their whole collection of posted tweets, even if other options of tags are available for similar purposes.

Table 2 shows more information on the hashtags of the dataset, including more detailed facts about the most frequent tags among female and male users. By dividing the number of tweets in which hashtags appeared by the number of users that employed hashtags, it becomes clear that, on average, men used slightly more tags than women in our dataset: 2.67 hashtags per male user versus 2.20 hashtags per female user. It is also important to note that the frequency of retweets - the re-posting of someone else's tweets - in this dataset is almost negligible. This is a meaningful information because significant rates of retweeting could affect the outcome of the experiment, since some public figures appeal more to men, others to women.

**Table 2:** Information on the most frequent hashtags of the dataset.

Hashtag		All users	
	number of tweets	number of users	% of retweets
#serra45	43,864	12,667	0.00%
#dilma13	27,887	7,873	0.00%
#brasil13	23,824	5,862	0.00%
#dia31vote13	17,028	4,673	0.00%
#13neles	16,802	6,708	0.00%
Total (all tags)	609,953	245,589	0.03%

		Female users	
	number of tweets	number of users	% of retweets
#serra45	14,525	4,866	0.00%
#dilma13	8,967	2,682	0.00%
#brasil13	8,282	2,032	0.00%
#13neles	6,290	2,730	0.00%
#dia31vote13	5,968	1,659	0.00%
Total (all tags)	212,459	96,628	0.01%

		Male users	
	number of tweets	number of users	% of retweets
#serra45	29,339	7,801	0.00%
#dilma13	18,920	5,191	0.00%
#brasil13	15,542	3,830	0.00%
#dia31vote13	11,060	3,014	0.00%
#13neles	10,512	3,978	0.00%
Total (all tags)	397,494	148,961	0.04%

#### Comparing Hashtag Usage

In this paper, we are interested in comparing discursive strategies used by men and women when designating hashtags to their political tweets. This task will be achieved by measuring the usage of certain hashtags by female and male users. Our working hypothesis is that there are indeed differences between the choices of hashtags by Brazilian men and women, since this kind of difference is also observed in offline interactions, as shown earlier in the paper.

However, due to the distinct total amount of messages generated by men and women, the respective raw participations of each gender in the adoption of a given hashtag are not directly comparable. For instance, simply stating that 60% of the usage of a particular hashtag comes from female members does not mean that women are the main adopters of this tag: if their participation in the complete subdataset is 70% - e.g. due to a massive support from female voters to a given candidate -, then we consider men as the main adopters of this specific tag.

In order to determine whether a particular tag is more common among users of a certain gender, z-score values were assigned to each tag. In this approach, z-scores operate as scaling factors so that parallels between genders can take place using a common measure of comparison.

Z-scores represent the distance, in terms of standard deviation units, between raw scores and the mean: negative z-scores indicate raw scores below the mean, while positive z-scores indicate raw scores above the mean. Z-scores can be calculated according to z = (*x*-*μ*)/*σ*, where *x* stands for the raw score (percentage of occurrences generated by female or male users for each hashtag), *μ* indicates the mean (percentage of occurrences generated by female or male users for the whole subdataset) and *σ* symbolizes the standard deviation. For example, if a given hashtag is more used by women than the entire subdataset from which it is part, then its ''female z-score'' - that indicates the correspondent weight of the female usage - is positive, and its ''male z-score'' - indicating the correspondent weight of the male usage - is negative. Thus, the use of z-scores avoids problems that arise from the different percentage of men and women in the subdatasets.

''Female'' and ''male z-scores'' are complementary, so, for a given hashtag, their sum is always equal to zero. As matter of convenience, all z-scores presented in this study are associated to the hashtag usage of female users (''female z-scores''). Therefore, positive z-scores will indicate a prevalence of female usage and negative z-scores will always indicate a prevalence of male usage.

## Results

As said before, studies of language and gender investigate the crucial yet often unnoticed role that gender - which is, unlike sex, a social construction rather than a biological determinant - plays in our daily linguistic behavior in relation to discursive strategies [19]. Reflections on these previously cited studies led us to hypothesize that, also in the context of the tag designation process, gender might act as a social factor able to influence the choice of a linguistic form. In this section, before discussing the results obtained in our analysis, we briefly present specific related work on gender and written style in offline and online scenarios.

First, it is important to mention that several approaches have been taken to the analysis of the relation between gender and speech [20]. In this work, we adopt the *difference* one [21], that considers female and male speakers as part of different subcultures that demand from each individual characteristic modes of expression according to the subculture to which he or she belongs.

The study of differences between female and male writing styles was performed by Argamon et al. [22], who found that, even in formal writing, women tend to use more features identified as ''involved'' - that typically show interaction between the speaker/writer and the listener/reader, such as first and second person pronouns - while men exhibit greater usage of features identified as ''informational'' - like noun specifiers and quantifiers. In the context of marital conflict, the use of computational tools showed significant distinctness between genders in some aspects, including in the higher female adoption of ''social words'', like those related to family and friends, which may reflect a female concern for others [23].

Dissimilarities between female and male language use were also found in computer-mediated communication [24] and in online environments, including during the task of Web searching [25]. According to Rossetti [26], men more often use e-mail discussion groups to extend their own influence and authority, and the determination of the gender of an e-mail's author, based on the gender-preferential language used, was implemented by Corney [27]. Disparities in writing style and content among bloggers of distinct genders were likewise observed: previous studies showed that, in this context, women tend to use a more personal writing style [28], but other findings also indicated that most of the differences between female and male bloggers are related to the social goals of the blog [29] and the genre of the texts published [30]. These results reveal that, in order to avoid bias in the conclusions, it is important to control the topic of the messages, so that textual differences due to distinctness of contexts will not be interpreted as social differences. Therefore, in our analysis we focus on messages within the political discourse in the Brazilian context.

Other studies illustrated gender differences in writing style in the ambience of online social networking systems. The analysis of female and male descriptions of images and albums in Pinterest highlighted that, in a general perspective, women are more prone to use terms that convey affection and men tend to employ expressions that assert their power and status [31]. Again, however, the context in which these descriptions are used - for example, to describe family albums or technology portfolios - may influence the type of discourse adopted. Burger et al. [15] used text features for the construction of a gender predictor for Twitter members; and Bamman et al. [32] analyzed gender as a social variable in Twitter messages - the authors demonstrated, for instance, the existence of multiple gendered styles in tweets. We, on the other hand, focus on discursive strategies linked to social roles carried out by users when using a particular feature of Twitter: the hashtags. This, by itself, brings an original perspective to our study.

Therefore, it is possible to summarize that the existing literature on language use in online environments is rich in studies that show linguistic differences between men and women on the Web. In general, it can be stated that men are more prone to employ more assertive linguistic strategies to reinforce their power in society [26], [31], while women tend to prefer the adoption of confidential strategies that do not put them in a position of authority over the interlocutors [22], [23], [31].

The main hypotheses that have emerged from the above mentioned studies on linguistics and language use in social media are the following: (a) there are differences between the ways men and women express their attitude toward a given candidate through the use of hashtags in Twitter; (b) as in other situations described before, female users prefer more confidential strategies, while male users tend to adopt more assertive ones. The vision of societies being polarized by power and solidarity [10], [11], cited earlier in this paper, also suggests that, in general, Western men are expected to use linguistic forms that assert their power to a general audience and that Western women are expected to adopt more neutral forms, so that they are not seen, by other members of the communities, as challenging male power. Although asymmetrical relationships between men and women are a changing scenario in most societies, many inequalities still remain and reflect on language use.

To verify our hypotheses, we inspected the hashtags in our subdatasets in order to find linguistic elements that could evidence distinct discursive strategies between men and women in Twitter.

Among the tags expressing some kind of support to Dilma and Serra, we were able to identify at least two groups of particular interest: (1) those focused on the user clearly informing his/her option for a candidate; and (2) those focused on the user suggesting/imposing a candidate for the readers. In group 1, we include the tags containing verbs inflected in the first person singular indicative mood, such as #votodilma (''I vote for Dilma'')/#votoserra (''I vote for Serra'') and #euquerodilma (''I want Dilma'')/#euqueroserra (''I want Serra''). In group 2, on the other hand, we include the tags containing verbs inflected in the second person singular imperative mood, expressing a command urging the audience to act a certain way, as in #vote13 (''Vote for 13'' [Dilma's number])/#vote45 (''Vote for 45'' [Serra's number]) and #sejamais1dilma (''Be one more for Dilma''). At this stage, we were also expecting to analyze hashtags expressing opposition to the candidates. However, among them, those which clearly and openly aimed to inform one's preference or to suggest readers not to vote in any of the candidates using one of the above linguistic strategies, like #naovotodilma (''I do not vote for Dilma'')/#naovoteserra (''Do not vote for Serra''), did not appear.

These discursive strategies, although both have ultimately the same goal - to express one's attitude toward a given candidate -, seek to achieve the target in two undoubtedly different ways. It is possible to understand the use of first person indicative mood forms as implying a balanced connection between the author and the reader, as though the former said ''I vote for candidate *x*, why don't you do it too?''. Still, the use of imperative forms may suggest a higher hierarchical position from which the author operates, as if he or she had some sort of power over the reader. Naturally, those implications - balanced connection between users and higher power from the author - are not necessarily real: they can be simple reflections of the roles expected to be played by certain individuals in offline situations.

**Figure 4:**
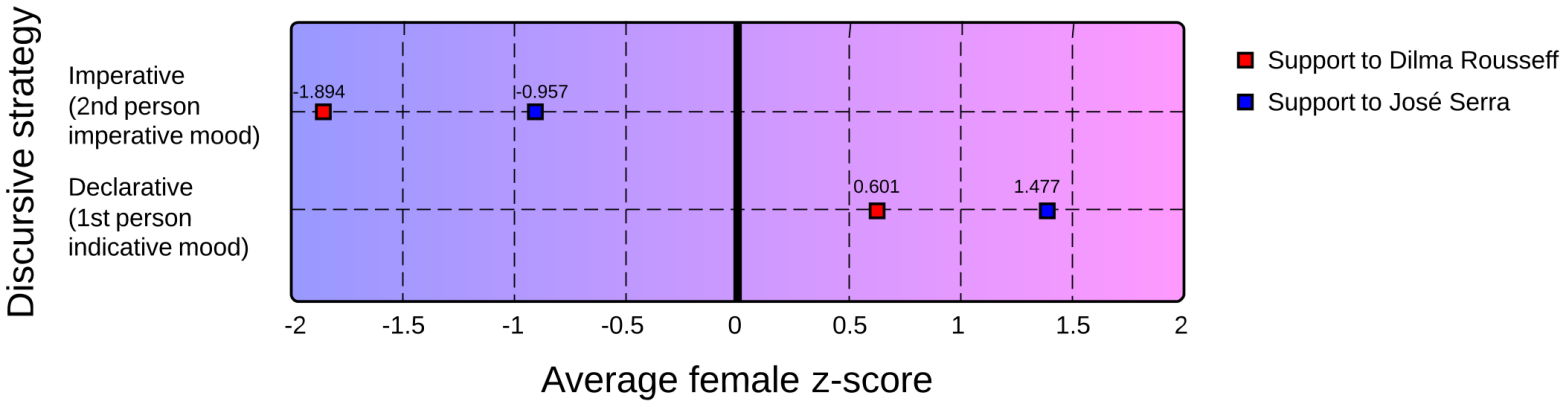
Average female z-scores of group 1 (''declarative tags'') and group 2 (''imperative tags''). Hashtags on group 2 are more used by men while those on group 1 are more common among women. Standard deviations are the following: -1.894±0.325, -0.957±0.424, 0.601±0.668, 1.477±0.574.

The computation of the average z-scores of the groups of hashtags associated to the two genders showed significant differences in the behavior of men and women expressing their attitude towards the candidates. The forms belonging to group 1, which brings tags with verbs in the first person singular indicative mood and that can be called ''declarative'' tags, tend to be more used by women. However, the hashtags containing verbs in the second person singular imperative mood from group 2, that can be considered ''imperative tags'', seem to be more common among male users. Figure 4 illustrates these differences and shows that these results are valid for supporters of both political candidates.

These results are partially consistent with our theoretical assumptions and seem to corroborate most of the aforementioned linguistic studies, especially those that indicate a male's propensity to manifest authority through language: men use more imperative - therefore more influential - hashtags, overtly expressing their intention of acting over followers, which can be interpreted as a sign of assertiveness and confidence in their own power.

Regarding the finding showing that women are the main adopters of the declarative hashtags, which are more informational as they simply state their political option, a superficial analysis could lead to the conclusion that there is a conflict between the result found and the theoretical assumptions, since it could be expected that men would also be the main adopters of these more objectively informational and self-directed forms [22], [23]. Nevertheless, a more refined level of analysis, considering the already mentioned polarization of societies in relations of power and solidarity [10], [11], proposes a more complex interpretation: because men are considered occupying more powerful positions in the social hierarchy of modern Latin American societies [11], they would feel more free to use such overtly influential forms like imperatives. Women, on the other hand, trying to avoid being in direct confrontation - that could be the case if they used imperative forms toward an audience occupying a higher social rank - prefer the declarative ones. For example, in a perspective which claims that both men and women are employing persuasive strategies, it becomes clear that each of them unconsciously chooses the one that is most compatible with the gendered social role expected to be performed.

Other perspectives of analysis shall likewise be taken into consideration. For instance, it is possible that some of the observed patterns in discourse could be related to in-group communication [33], that is, intended for social support and reinforcement among people who already support the same candidate. In this case, hashtags should be seen as signals of users' membership to given groups. Another possibility is that the observed phenomena are the result of mimetic processes, given that, in case female and male users cluster together in the network, they may be exposed to different hashtags. Since our dataset does not provide following links among users, but only tweets and profile information, we were not able to verify exposure on individual levels or to check for gendered clusters.

The results found here are also related to previous research in the fields of psychology, anthropology, communication and discourse analysis, that showed differences in the ways Western men and women try to convince others and are persuaded by them [34], [35], even in computer-mediated environments [36]. It was found that men tend to feel more confident in their own skills to persuade [37] and this may be the reason that makes them more comfortable to use more straightforward discursive strategies, such as imperative hashtags in group 2. It was also said that public persuasion is a predominantly masculine practice in Western societies [38]. Other studies pointed that, given some conditions, Western women are more easily influenced and less influential than men [39], which leads to questions such as ''what types of behavior do people use when trying to influence men or women'' [40]? In our study, we identify what could be one of these behaviors in an unexplored situation so far: although these results are not new in the process of human communication, this is, to the best of our knowledge, the first time that they were observed in the domain of online social networking communication and related to the use of tags in a completely free tagging environment. However, it is important to be clear that different behaviors regarding persuasive strategies are not directly linked to sex, but to power and status, so that gender differences in behavior must be understood within a broader context of social relations [41].

Our results can also be analyzed from a political perspective. The largest negative value for the average female z-scores among the imperative tags and the smallest positive value among the declarative tags posted by supporters of Dilma Rousseff indicate that her voters are more prone to use the imperative hashtags than supporters of José Serra, who prefer, in general, the declarative discursive strategy.

## Discussion

As Tannen [19] suggests, conducting research on gender is like stepping into a maelstrom, since variables are so many and so complex - and, in our opinion, also because scientific research on gender has the potential to reify and reproduce existing prejudices and inequalities. As a result, one of the aspects of this type of research is its interdisciplinary nature, whereas only approaches that bring together points of view from different fields of knowledge are able to comprehend and properly explain phenomena such complexly built. Thus, studies that attempt to delineate differences in profile and behavior of online users are enriched by studies that examine the linguistic behavior of these users. This collaboration is important to introduce new directions and ideas for improving research and to identify questions that scholars working merely in their respective fields would not have asked without exchanging information with colleagues from other areas.

This work proposes and presents an innovative gender based analysis of the tag designation process in a social networking service, differing from previous ones in that it considers gender as a social factor that might influence the choice of a specific tag among those related to a given topic. We aim to verify whether the already known difference in the linguistic behavior of men and women also occur in the communication across online social networks and, more specifically, during the task of tagging in social media. In order to perform this analysis, we concentrate on data collected from Twitter and, in particular, we examine the use of hashtags in tweets. Our results suggest that, at least on the level of discursive strategies, this distinctness does exist and it can be quantified, as we did when analyzing the different political attitudes adopted by female and male Brazilian Twitter users. We reckon that this investigation adds an interesting new dimension to the study of language use in social media and to the investigation of human tagging behavior, since we propose the adoption of a dataset of hashtags as a corpus for linguistic research - which is methodologically quite different from the usage of full tweets.

Our outcomes, rather than just indicate specific situations in which gender plays important roles in communicative situations, serve to provide foundations and to foster research in the field of Internet linguistics. According to our view, it is particularly important to encourage and promote empirical research combined with social theoretical analysis: the qualitative study of big data collected from social media, which changed the possibilities of investigating human attitude in society [42] and created opportunities to study social and cultural processes and dynamics in new ways [43], is interesting to linguistic studies since it makes it possible to understand the behavior of individuals and communities in an increasingly relevant social environment and allows the discovery of correlations and differences between online and offline speeches.

The findings presented here can be useful for computational tasks such as developing tag recommendation systems based on users' collective preferences, tailoring targeted advertising strategies, identifying followers of a given political trend and recognizing political bias in information networks. For recommendation and personalization purposes, however, a critical discussion on how computer technology may reinforce existing differences between mainstream enactments of female and male genders seems to be crucial. Future work may include the use of machine learning algorithms to automatically obtain the classification of the subdatasets, the investigation of the role of other social factors - like age and location - in tagging behavior and the analysis of other discursive strategies adopted by men and women in different online situations.
